# Early results of the Resilia Inspiris aortic valve in the old age patients - a retrospective comparison with the Carpentier Edwards Magna Ease

**DOI:** 10.34172/jcvtr.2020.38

**Published:** 2020-08-26

**Authors:** Magjun Shala, Lars Niclauss

**Affiliations:** ^1^Faculty of Medicine and Biology, Lausanne University, Lausanne, Switzerland; ^2^Cardiovascular Department, Division of Cardiovascular Surgery, University Hospital Lausanne (CHUV), Lausanne, Switzerland

**Keywords:** Surgical Aortic Valve Replacement, Inspiris Resilia Aortic Valve, Tissue Preservation, Bioprosthetic Aortic Valve, Hemodynamics

## Abstract

***Introduction:*** The Inspiris Resilia aortic valve® (INSPIRIS) is a pericardial bio-prosthesis with a new sterilization procedure that shows promising results in terms of reduced calcification.

***Methods:*** The 30-day mortality and morbidity were analyzed, comparing the INSPIRIS implanted between May 2017 and the end of January 2019, with its "predecessor", the Carpentier-Edwards Perimound Magna Ease (ME). Echocardiography was performed one-week after surgery. 125consecutively operated patients were included (59 INSPIRIS, 66 ME).

***Results:*** One patient in the ME group died and one patient in the INSPIRIS group had a complicated postoperative course due to right heart failure. Two patients (one INSPIRIS, one ME patient) suffered a perioperative stroke. The hemodynamic evaluation shows an effective reduction of mean transvalvular pressure gradients after surgery in both groups. INSPIRIS tended to have lower trans-prosthetic pressure gradients (9 mm Hg, Interquartile range [IQR] 11-7 mm Hg versus 12 mm Hg, IQR 15-9 mmHg; P = 0.001), reduced trans-prosthetic blood flow acceleration (209 cm/s, IQR 220-190 cm/s versus227 cm/s, IQR 263-191 cm/s; P = 0.003) and increased permeability indices (57%, IQR 67%- 47% versus42%, IQR 48%-38%; P8%; P < 0.001).

***Conclusion:*** There are only few clinical data available from INSPIRIS, and the present analysis confirms good results initial postoperatively with a tendency towards possibly improved hemodynamics compared to ME.

## Introduction

Surgical aortic valve replacement (SAVR) as an isolated or combined procedure remains a treatment option for degenerative valve disease. Current research focuses on new preservation technologies for xeno-pericardial prostheses with the aim of improving long-term durability, lowering the age threshold for bio-prosthetic valve implantation and thus reducing the side effects of long-lasting anticoagulation.^1^ The increasing shift of an indicated age threshold for bio-prosthetic valve implantation towards younger patients may also lead to new long-term complementary treatment concepts, especially in times of success of the trans-catheter aortic valve implantation (TAVI).

The Inspiris Resilia aortic valve® (INSPIRIS) (Edwards Lifesciences LLC, Irvine, USA) is a new bovine pericardial prosthesis for SAVR. The functional group capping of aldehydes (glutaraldehyde-fixed tissue) to prevent oxidation and calcification, glycerolization to reduce the amount of residual chemicals and the new sterilization process in which the water of the pericardial tissue is displaced and replaced by glycerol (which allows dry storage) are the key innovations that promise lasting tissue integrity. In vivo animal models confirmed significantly reduced calcifications and initial clinical studies showed promising follow-up results.^2-4^

The use of INSPIRIS for SAVR, especially in younger patients, can combine the advantages of a durable bio-prosthesis with the potential future option of a simplified valve-in-valve procedure for late structural valve deterioration by incorporating an extendable stent for smaller sized annular diameters. The INSPIRIS has been available on the European market since the spring of 2017 after receiving the CE mark. Post-operative clinical data are therefore rare.

We present a small series of early postoperative results (morbidity and mortality) of the INSPIRIS and compare them with those of its “predecessor”, the Carpentier-Edwards Perimound Magna Ease (ME) (Edwards Lifesciences LLC, Irvine, USA) aortic bio-prosthesis, with a special emphasis on a comparative evaluation of their postoperative hemodynamic performance.

## Materials and Methods

A single database of two surgeons was reviewed, retrospectively searching for either isolated or combined aortic valve replacement with INSPIRIS. The data collection started in February 2019 and covered the period from the beginning of the INSPIRIS implantation in May 2017 until the end of January 2019. According to these 21 months of observation, a comparison group was formed. This control group consisted of patients who had been operated on by the same team during the 21 preceding months (August 2015 to April 2017) and who had thus received the predecessor model, the ME.

Included were elective and urgent, isolated or in combination with coronary artery bypass graft (CABG) performed aortic valve replacements. Patients with concomitant mitral valve or aortic root surgery as well as patients with severe pre-existing left ventricular (LV) dysfunction were excluded with the aim of at least partially reducing possible factors that might influence the postoperative hemodynamic performance of the prostheses to allow for better comparability of the groups.

All patients had a postoperative echocardiography after one week. In addition to the left ventricular ejection fraction (LVEF), the mean pressure gradients (mm Hg), blood flow accelerations (cm/s) and permeability indices (%) of the bio-prostheses were analyzed. Clinical follow-up after 30 days was completed for all patients and early postoperative mortality and morbidity were documented. The latter was classified into major complications, i.e. non-fatal myocardial infarction, early re-operation (pericardial tamponade, deep sternal wound infection, etc), endocarditis and stroke, or into minor complications including new postoperative arrhythmias requiring permanent pacemaker implantation (PPM), acute renal failure and blood transfusions. All-cause mortality, postoperative morbidity and postoperative hemodynamic parameters of the two different prostheses were compared. The primary endpoint was to demonstrate the non-inferiority of the INSPIRIS.

### Statistical analysis

The SPSS 22.0 software package for Windows (SPSS Inc. Chicago, Illinois, USA) was used. Categorical variables are presented as numbers and proportions (%). Continuous normally distributed variables are summarized as mean ± one standard deviation (SD), while non-normally distributed variables (according to the Kolmogorov-Smirnovand the Shapiro-Wilk test) are described by their median and interquartile range (IQR). Accordingly, either the *t* test or the Mann-Whitney U test was used to compare the results. A *P* value <0.05 was considered significant. The authors had full access to the data and take responsibility for its integrity.

## Results

### Demographics, risk and operative data

125 patients, 59 in the INSPIRIS group and 66 in the ME group, were identified. All were operated on during the observation period and met the inclusion criteria. The main demographic parameters (age, gender, etc), risk factors and existing comorbidities as well as the logistic EuroSCORE 2 risk calculations were comparable (Table 1, section A). The indications for surgery and the number of single versus combined procedures were similar, except for an increased number of bicuspid valves in the ME group (Table 1, section B). More ME patients were anticoagulated after surgery. This is mainly due to the different observation period in which the operations were performed. Systematic postoperative anticoagulation was only gradually abandoned in favor of the recommended platelet anti-aggregation in recent years (Table 1, section B).

**Table 1:** Demographics, surgical risk, clinical + echo outcome

	**INSPIRIS** **n = 59**	**Magna Ease** **n = 66**	***P*** **value**
**A) Demographic parameters/Risk factors**	**Mean ± SD / No (%)**		
Age (years)	71 ± 7	69 ± 9	0.15
Gender (female)	15 (25)	22 (33)	0.33
Body mass index (kg/m^2^)	28 ± 5	28 ± 5	0.95
Obesity	15 (25)	21 (32)	0.38
Previous/current tobacco abuse	22 (37)	28 (42)	0.99
Hypertension	44 (75)	42 (64)	0.13
Dyslipidemia	30 (51)	33 (50)	0.7
Chronic obstructive pulmonary disease	4 (7)	4 (6)	1
Insulin dependent diabetes	6 (10)	2 (3)	0.14
Non-insulin dependent diabetes	11 (19)	7 (11)	0.13
Chronic renal failure (all without dialysis)	2 (3)	6 (9)	0.3
Peripheral artery disease	6 (10)	2 (3)	0.15
Significant coronary disease (concomitant CABG)	25 (42)	27 (41)	0.91
EuroScore (%)	2.34 ± 1.6	2.82 ± 2.8	0.27
**B) Aortic disease/Type of surgery**	**Median [IQR**_3/4-1/4_ **] / No (%)**		
Aortic stenosis	48 (81)	56 (85)	0.6
Aortic regurgitation	11 (19)	10 (15)	0.6
Bicuspid aortic valve	10 (17)	23 (35)	0.02
Implanted valve size	23 [25-23]	23 [25-23]	0.51
***Type of operation***			
- Isolated AVR	39 (66)	43 (65)	0.91
- AVR + concomitant coronary bypass surgery	20 (34)	23 (35)	0.97
***Concomitant procedures ***			
- Septal myectomy	16 (27)	10 (15)	0.1
- Ascending aortic replacement / reduction plasty	18 (31)	23(35)	0.56
- Left atrial appendage ablation	2 (3)	5 (8)	0.45
***Operation times (min)***			
- Aortic cross-clamping	72 [97-55]	82 [108-64]	0.06
- Cardiopulmonary bypass	92 [133-80]	103 [128-86]	0.09
Post-operative anticoagulation	19 (32)	45 (68)	**<0.001**
**C) Clinical outcome**	**Median [IQR**_3/4-1/4_ **] / No (%)**		
Atrial fibrillation / Flutter	20 (34)	27 (41)	0.42
Planed PPM implantation for preoperative rhythmic disorders	2 (3)	1 (1.5)	0.60
New conduction disorders	**-**	**-**	**-**
Acute renal failure-no dialysis	6 (10)	4 (6)	0.52
Transfusion (red cell concentrate)	7 (12)	4 (6)	0.52
In-hospital mortality (= cardiac related death)	-	1 (2)	1
Perioperative myocardial infarction	**-**	**-**	**-**
***Re-operation***	8 (14)	8 (12)	0.81
- Tamponade	5 (8)	7 (11)	0.69
- Mediastinitis	2 (3)	1 (2)	0.60
- Sternal opening for right ventricular dysfunction	1 (3)	**-**	**-**
Stroke	1 (2)	1 (2)	1
Minor neurological disorders (Agitation/disorientation)	6(10)	5 (8)	0.61
ICU stay, days	1 [2-1]	1 [2.3-1]	0.79
**D) Echocardiographic outcome**	**Mean ± SD / Median [IQR**_3/4-1/4_ **]**		
***Postoperative all***	***n = 59***	***n = 66***	
Postoperative mean gradient (mm Hg)	10 [11-7]	12 [15-9]	**<0.001**
Postoperative V max (cm/s)	210 [232-196]	226 [262-192]	**0.008**
Permeability index (%)	54 [65-47]	42 [48-38]	**<0.001**
LVEF (%)	60 [60-55]	60 [67-50]	0.25
***Postoperative aortic stenosis***	***n = 48***	***n = 56***	
Postoperative mean gradient (mm Hg)	9 [11-7]	12 [15-9]	**0.001**
Postoperative V max (cm/s)	209 [220-190]	227 [263-191]	**0.003**
Permeability index (%)	57 [67-47]	42 [48-38]	**<0.001**
LVEF (%)	60 [61-55]	62.25 [68-55]	0.15
***Preoperative aortic stenosis ***	***n = 48***	***n = 56***	
Indexed preoperative surface area (cm^2^/m^2^)	0.47 ± 0.19	0.42 ± 0.2	0.38
Preoperative mean gradient (mm Hg)	40 [56-32]	40 [54-26]	0.39
***Mean gradient reduction ***	33 [46-24]; *P* < 0.001*	26 [42-15]; *P* < 0.001*	0.17

P*-refers to the direct comparison of the pre- versus postoperative mean gradients in aortic stenosis, SD-Standard deviation, IQR-Interquartile range = quartile 75%-quartile 25% (Q3-Q1), AVR-Aortic valve replacement, PPM-Permanent pacemaker, ICU-Intensive care unit, V max – Maximum velocity, LVEF-Left ventricular ejection fraction.

### Mortality and Morbidity

One ME patient died, probably due to an annular disruption that led to massive right ventricular intramural bleeding. After initial stabilization, successful hemostasis and admission to the intensive care unit (ICU), the patient had a cardiac arrest a few hours later, probably due to a severe right ventricular dysfunction (Table 1, section C).

One INSPIRIS patient also had a complicated postoperative course. After surgery (aortic valve replacement and CABG), he developed severe right ventricular (RV) dysfunction requiring urgent reopening of the sternum. A coronary angiography confirmed patent grafts. The patient remained unstable and started bleeding again from the sternum due to a beginning disseminated intravascular coagulation. After blood transfusions and sternal wound revisions with a “chest packing”, the patient gradually stabilized and improved his RV function. The sternum was closed 10 days later and the patient left the hospital after one-month. 17 months later he was still alive (as he was admitted to hospital for other non-cardiac complaints).

In two patients from the INSPIRIS and one from the ME group, epicardial PPMs were implanted simultaneouslyduring surgery, which were indicated due to pre-operative rhythmic disorders. Nobody required PPM implantation due to new, procedure-related conduction disorders. Deep sternal wound infections requiring reopening of the sternum with subsequent vacuum-assisted closure and rewiring occurred in two patients from the INSPIRIS group and one patient from the ME group. No postoperative endocarditis (infection of the valve prosthesis) was observed.

A symptomatic perioperative stroke occurred in one INSPIRIS and one ME patient. Both showed a new right hemiparesis immediately after surgery with confirmed acute cerebral ischemic lesions at MRI. The clinical course was favorable in both patients, with a progressive recovery.

### Hemodynamic evaluation after 1 week

In both groups a mean trans-prosthetic pressure gradient reduction (INSPIRIS 33 mmHg, IQR 46-24 mm Hg versus to ME 26 mm Hg, IQR 42-15 mm Hg; *P* = 0.17) of the stenotic aortic valves was achieved.Echocardiography showed slightly reduced mean trans-prosthetic pressure gradients, reduced trans-valvular flow accelerations and increased permeability indices in favor of INSPIRIS (Table 1, section D).

To obtain a reliable comparison, only patients with severe native valve stenosis were compared. INSPIRIS still showed lower mean trans-prosthetic pressure gradients (9 mm Hg, IQR 11-7 mm Hg versus 12 mm Hg, IQR 15-9 mm Hg; *P* = 0. 001), reduced trans-prosthetic blood flow acceleration (209 cm/sec, IQR 220-190 cm/s versus 227 cm/s, IQR 263-191 cm/s; *P* = 0.003) and increased permeability indices (57%, IQR 67-47% versus 42%, IQR 48-38%; *P* < 0.001).

The comparison of the postoperative hemodynamic results of the two prostheses regarding their different sizes (for preoperative stenotic valves) showed significantly lower mean gradients in favor of 21 and 25, reduced flow acceleration in favor of 25 and increased permeability indices in favor of all small and medium INSPIRIS compared to ME (Table 2).

**Table 2:** Detailed postoperative hemodynamic evaluation for aortic stenosis

		**INSPIRIS**	**Magna Ease**	***P*** **value**
		**n = 48**	**n = 56**	
		**Median [IQR**_3/4-1/4_ **]**		
**Postoperative LVEF (%)**	**60 [61 - 55]**	**62.25 [68 - 55]**	**0.15**
Annular size (mm)	21	65.5 [67 - 63]	69 [70 - 67]	0.27
	23	60 [64 - 55]	60 [65 - 55]	0.66
	25	60 [60 - 55]	62.5 [66 - 50]	0.23
**Post-op mean gradient (mmHg)**	**9 [11 - 7]**	**12 [15 - 9]**	**0.001**
Annular size (mm)	21	10 [12 - 9]	14.5 [16 - 13]	0.03
	23	11 [11 - 7]	10.5 [15 - 9]	0.16
	25	8 [9 - 8]	13 [15 - 8]	0.003
**Post-op V max (cm/s)**	**209 [220 - 190]**	**227 [263 - 191]**	**0.003**
Annular size (mm)	21	222 [241 - 207]	257 [267 - 229]	0.11
	23	209 [216 - 197]	220 [247 - 204]	0.08
	25	203 [218- 184]	228 [262 - 184]	< 0.05
**Permeability index (%)**	**57 [67 - 47]**	**42 [48 - 38]**	**< 0.001**
Annular size (mm)	21	52.5 [55 - 50]	39 [41 - 38]	0.036
	23	55 [63 - 47]	44.5 [49 - 41]	0.004
	25	57 [71 - 50]	44 [50 - 38]	0.004

LVEF - Left ventricular ejection fraction, V max - Maximum velocity. Annular size of 27 was almost exclusively implanted in patients with moderate to severe aortic insufficiency.

## Discussion

Based on these early postoperative results, comparing INSPIRIS with its predecessor model, the ME, it can be concluded the prosthesis is safe. Postoperative mortality and morbidity were low in both successively operated comparable groups. By including high-risk patients and patients with combined procedures, a cross-section of the patient population currently assigned to the SAVR was obtained.

Currently, only few postoperative data are available from INSPIRIS. Bartuś et al reported their experience with 133 INSPIRIS patients, the vast majority of whom (≈86%) had isolated SAVR, with full follow-up after one year being about 90%. The study was designed as a prospective safety study and was sponsored by the manufacturer before INSPIRIS was launched on the European market.^4^ Based on their findings, the authors concluded that Inspiris was safe due to the observed low complication rate and had acceptable hemodynamic performance.^4^ This last point was supported by indirect comparison with the hemodynamic properties of various predecessormodels evaluated in previous studies.^4^

In contrast, the present analysis focuses on a direct comparison of consecutive patients operated on by the same surgeons. A good postoperative hemodynamic performance with significantly reduced mean trans-aortic gradients was confirmed for both valve types. In addition, a tendency towards improved hemodynamic flow behavior in favor of INSPIRIS was observed, which was also at least partially confirmed by the direct comparison of the different implanted prosthesis sizes. Although this small difference in trans-valvular gradients (of 2 mm Hg and 3 mm Hg for stenotic valves) between the two study groups was statistically significant, the resulting potential clinical effects may be negligible.

Major limitations result from the non-randomized, retrospective approach, the limited number of patients and the short follow-up time. On the other hand, the fact that these (selected) patients were operated on by the same two surgeons using the same technique and within the same hospital during an equivalent timespan speaks for a certain comparability of the data. In this sense, the present results should be seen as an incentive to initiate further comparable and larger studies to analyze whether a potential hemodynamic advantage of INSPIRIS is reproducible and maintained during a longer follow-up.

## Competing interests

None declared.

## Ethical approval

Ethics approval was guaranteed according to international recommendations by the institutional review board of the investigator center (ethics no. 2018-02182).

## Funding

None.
